# Prestin Regulation and Function in Residual Outer Hair Cells after Noise-Induced Hearing Loss

**DOI:** 10.1371/journal.pone.0082602

**Published:** 2013-12-20

**Authors:** Anping Xia, Yohan Song, Rosalie Wang, Simon S. Gao, Will Clifton, Patrick Raphael, Sung-il Chao, Fred A. Pereira, Andrew K. Groves, John S. Oghalai

**Affiliations:** 1 Department of Otolaryngology–Head and Neck Surgery, Stanford University, Stanford, California, United States of America; 2 Bobby R. Alford Department of Otolaryngology – Head and Neck Surgery, Baylor College of Medicine, Houston, Texas, United States of America; 3 Department of Otolaryngology–Head and Neck Surgery, Chosun University, Gwangju, South Korea; 4 Department of Bioengineering, Rice University, Houston, Texas, United States of America; 5 Department of Neuroscience, Baylor College of Medicine, Houston, Texas, United States of America; 6 Department of Molecular and Human Genetics, Baylor College of Medicine, Houston, Texas, United States of America; 7 Program in Developmental Biology, Baylor College of Medicine, Houston, Texas, United States of America; University of Southern California, United States of America

## Abstract

The outer hair cell (OHC) motor protein prestin is necessary for electromotility, which drives cochlear amplification and produces exquisitely sharp frequency tuning. Tecta^C1509G^ transgenic mice have hearing loss, and surprisingly have increased OHC prestin levels. We hypothesized, therefore, that prestin up-regulation may represent a generalized response to compensate for a state of hearing loss. In the present study, we sought to determine the effects of noise-induced hearing loss on prestin expression. After noise exposure, we performed cytocochleograms and observed OHC loss only in the basal region of the cochlea. Next, we patch clamped OHCs from the apical turn (9–12 kHz region), where no OHCs were lost, in noise-exposed and age-matched control mice. The non-linear capacitance was significantly higher in noise-exposed mice, consistent with higher functional prestin levels. We then measured prestin protein and mRNA levels in whole-cochlea specimens. Both Western blot and qPCR studies demonstrated increased prestin expression after noise exposure. Finally, we examined the effect of the prestin increase *in vivo* following noise damage. Immediately after noise exposure, ABR and DPOAE thresholds were elevated by 30–40 dB. While most of the temporary threshold shifts recovered within 3 days, there were additional improvements over the next month. However, DPOAE magnitudes, basilar membrane vibration, and CAP tuning curve measurements from the 9–12 kHz cochlear region demonstrated no differences between noise-exposed mice and control mice. Taken together, these data indicate that prestin is up-regulated by 32–58% in residual OHCs after noise exposure and that the prestin is functional. These findings are consistent with the notion that prestin increases in an attempt to partially compensate for reduced force production because of missing OHCs. However, in regions where there is no OHC loss, the cochlea is able to compensate for the excess prestin in order to maintain stable auditory thresholds and frequency discrimination.

## Introduction

Outer hair cells (OHCs) amplify vibrations of the basilar membrane through high-speed changes in cell length, termed electromotility. Prestin, the motor protein in the lateral wall of OHCs, generates the force of electromotility [1] and the lack of functional prestin in either prestin knock-out or prestin 499 knock-in mice results in the absence of electromotility and severe hearing loss [2], [3]. Many factors can modulate prestin function, alter electromotility, and thus change measures of cochlear function, including membrane lipid tension and cholesterol content [4]–[6], intracellular anion concentration [7]–[9], and salicylate administration. Moreover, chimeric prestin mutant mice, in which some OHCs have normal prestin and some have no prestin demonstrate elevated auditory thresholds [10]. However, another mouse model in which the density of prestin in every OHC was reduced to 34% of normal did not show threshold changes in the low-mid frequencies, even though the OHC non-linear capacitance, a measure of functional prestin, was reduced [11]. Thus, the link between prestin levels, prestin function, and hearing remains unclear.

In an effort to further study the role of prestin regulation in cochlear function, we turned to a mouse model of human hearing loss caused by the Tecta^C1509G^ point mutation [12]. This mutation affects the α-tectorin protein and results in tectorial membrane malformations so that the tectorial membrane is shortened and only contacts the first row of OHCs in heterozygotes and does not contact any OHCs in homozygotes [13]. In studying this transgenic mouse, we found that both genotypes also have increased OHC prestin levels that results in larger electrically-evoked movements of the reticular lamina and greater otoacoustic emissions [14]. The prestin increase was surprising because α-tectorin is expressed in supporting cells and spiral limbus cells, but not in OHCs [15], [16]. Thus, we believed it unlikely that the point mutation directly affected prestin production.

Since both genotypes had hearing loss, we considered it possible that prestin expression was increased as part of a systems-level attempt to compensate for the hearing loss. If true, it is possible that prestin expression should also increase in other models of hearing loss. Here, we tested this hypothesis using a mouse model of noise-induced hearing loss. Our goal was to kill some OHCs with noise exposure and then correlate prestin levels in the residual OHCs to *in vivo* measures of cochlear function including auditory thresholds and basilar membrane tuning. We found that functional prestin levels did increase in residual OHCs, and this may represent one mechanism by which hearing can recover after injury.

**Table:** Table1. Primers for qRT-PCR.

Prestin	5′-CGACTTGTATAGCAGCGCTTTAAA-3′
	5′-TTCTTCTCGCTCCCATAATGAGT-3′
Myosin VIIa	5′-TGGTACACTTGACACTGAAGAAAAAGT-3′
	5′-CCATCGTTCAGCCTCTTGGT-3′
GAPDH	5′-CTTCGATGCCGGGGCTGGCATT-3′
	5′-TGTTGGGGGCCGAGTTGGGATAGG-3′

## Materials and Methods

### Animals

Mice were used in accordance with our experimental protocol that was approved by Institutional Animal Care and Use Committee at Stanford University. We used 5 to 6 week old wild-type CBA/CaJ mice for all experiments. These mice have stable auditory thresholds from post-natal day 21 through 12 months of age [17], [18]. Mice were anesthetized using ketamine (100 mg/kg) and xylazine (10 mg/kg).

### Noise exposure

The noise exposure method was fully described in detail [19]. Briefly, a custom-built box contained six piezo horns (TW-125, Pyramid Car Audio, Brooklyn, NY, USA) inserted through the cover. Band-passed white noise (4–22 kHz) was generated digitally with RPvds software (Version 6.6, Tucker-Davis Technologies, Alachua, FL, USA), converted to analog by a digital-to-analog converter, and then transferred to the power amplifier (Servo 550, Sampson, Hauppauge, NY, USA) to drive the speakers. A cage containing the mice was placed inside the box and the mice were exposed to noise at 98dB ±2 dB for 4 hours.

### Auditory brainstem responses (ABRs), distortion product otoacousticemissions (DPOAEs), and cochlear microphonics (CMs)

ABRs, DPOAEs, and CMs were measured as previously described [20]. ABRs and DPOAEs were serially measured in a cohort of mice before noise exposure as control (control) and at day 0.5 (0.5d), day 3 (3d), day 7 (7d) and 1 month (1 m) after noise exposure. Briefly, the ABR potentials were measured from needle electrodes positioned at the bottom of the tympanic bulla and at the vertex of the head, with a ground electrode placed in the rear leg. A bioamplifier (DP-311, Warner Instruments, Hamden, CT, USA) was used to amplify the signal 10,000 times. The sound intensity level was raised in 10 dB steps from 10 to 80 dB SPL and the sound frequency was varied between 4 to 46 kHz. At each sound level, 260 responses were sampled and averaged. The peak-to-peak value of the ABR was measured and the threshold at each frequency was calculated to be when this value was five standard deviations above the noise floor. If an ABR response was not detected at 80 dB SPL, we arbitrarily set the threshold to be 80 dB SPL for averaging purposes.

DPOAEs were measured by a probe tip microphone (type 4182, Brüel & Kjaer, Denmark) in the external auditory canal. The frequency response of this microphone was measured using a free-field microphone with a flat frequency response out to 100 kHz (type 4939, Brüel & Kjaer). This calibration curve was then used to adjust the DPOAE amplitudes we measured during the experiments. The sound stimuli for eliciting DPOAEs were two 1 second sine-wave tones of differing frequencies (F2 = 1.22×F1). We varied the range of F2 from 4 to 46 kHz. The two tones were of equal intensities and stepped from 20 to 80 dB SPL in 10 dB increments. The amplitude of the cubic distortion product was measured at 2*F1–F2. The threshold at each frequency was calculated to be when the DPOAE was >5 dB SPL and two standard deviations above the noise floor. If a DPOAE was not detected at 80 dB SPL, we arbitrarily set the threshold to be 80 dB SPL for averaging purposes.

The CM was measured after securing the mouse in a head holder and surgically opening the bulla to expose the round window. An ear bar to present the sound stimuli was then inserted into the ear canal and secured. A Teflon-coated silver wire with a ball-ended tip was placed on the round window membrane while a reference silver wire was inserted under the skin near the vertex of the skull. The ground electrode was placed in the hind leg. The stimuli were 30 ms 6 kHz tones with intensities ranging from 30–95 dB SPL. The CM signal measured by the bioamplifier was digitized at 500 kHz, and the magnitude of the response at 6 kHz determined by FFT.

### Patch clamping

One month old mice were sacrificed by cervical dislocation and their bullas were removed for further dissection in external solution containing 150 mM NaCl, 2 mM KCl, 2.5 mM MgCl_2_, 4 mM CaCl_2_, 10 mM HEPES, 5 mM glucose, 2 mM creatine, 2 mM ascorbate, 2 mM pyruvate, titrated with NaOH to pH 7.4. The final osmolality was 305–310 mOsm/kg. After removing some of the otic capsule bone, a small strip of the organ of Corti from the apical region about a half turn down from the helicotrema measuring 2–4 mm in length was dissected out and the tectorial membrane was gently brushed off. The epithelium was then secured by two strands of dental floss in a recording chamber filled with external solution. The experimental chamber holding the preparation was transferred to an upright microscope (Axioskop 2, Zeiss, Germany) and viewed through a 63X water-immersion objective.

Patch pipettes were made with a pipette puller (P1000, Sutter Instruments, Novato, CA) with initial resistances of 3–4 MΩ. The standard internal solution contained 5 mM EGTA, 135 mM KCl, 3 mM MgCl_2_, 3 mM ATP, 5 mM phosphocreatine, 10 mM HEPES, 2 mM ascorbate, titrated with NaOH to pH 7.25. The final osmolality was 290–295 mOsm/kg. OHCs were whole-cell voltage clamped with an Axon 200B amplifier using jClamp software (SciSoft, CT). Only cells with resting potentials <−50 mV were studied further. First, currents were measured in response to a voltage step protocol. Next, cell capacitance was measured using a continuous high-resolution two-sine wave stimulus protocol superimposed onto a voltage ramp from −150 to +120 mV [21]. Capacitance data were fit to the first-derivative of a two-state Boltzmann function using the software Igor Pro 6.0 (WaveMetrics, Lake Owego, OR) to calculate total charge transfer (Q_max_), the voltage at peak capacitance (V_1/2_), and the slope factor of the voltage dependence (α) [22]:
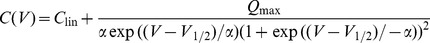
with C_lin_ calculated as the average capacitance at −150 mV and +120 mV. The charge density was then calculated using the specific membrane capacitance previously determined for mouse OHCs [23]. The following equation was used:



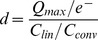
where d is charge density or the number of electrons moved per square micrometer of cell surface area, e^−^ is the electron charge (1.602×10^−19^ C), C_lin_ is the linear capacitance, and C_conv_ is the specific membrane capacitance of 0.008 pF/µm^2^.

### Basilar membrane vibratory tuning curves

Basilar membrane (BM) vibrations were measured using a custom-built optical coherence tomography system as previously described [24]. Briefly, mice were anesthetized with a ketamine/xylazine mixture, secured in a head holder, and the bulla was surgically opened. The mouse was oriented to view the region of the cochlea one half turn down from the helicotrema. By imaging non-invasively through the otic capsule bone, vibratory data were collected from the mid-portion of the basilar membrane over the frequency range from 3 to 12 kHz. For each frequency, we averaged 16trials using a stimulus intensity of 60 dB SPL. The resonance frequency was defined as the frequency at which the maximum vibratory amplitude was found (always between 8–9 kHz in this preparation). The response of the ossicular chain was also recorded to sound stimuli from 3 to 12 kHz at 80 dB SPL and scaled linearly to normalize the basilar membrane vibratory measurements. The frequency range was normalized to the resonance frequency to allow averaging between different mice. Q_10dB_ values were calculated as the resonance frequency divided by the bandwidth 10 dB down from the peak.

### Compound action potential (CAP) tuning curves

Masked auditory nerve tuning curves (also called CAP tuning curves) were measured in mice based on a routine technique [25], [26]. Data acquisition was performed using custom software programmed in MATLAB (Release 13, The Mathworks, Natick, MA). One speaker provided the probe tone and another provided the masking tone. The probe lasted 3 ms with a repetition time of 42.7 ms. Electrical responses to the probe tone were measured using the same subcutaneous needle electrodes and bioamplifier used for ABR measurement. Two hundred and sixty ABR responses were averaged. Initially, the masking tone was turned off and the probe amplitude was adjusted to the level needed to obtain a 1.2 µV N1/P1 response (i.e. the CAP originating from the auditory nerve fibers). Next, the masking tone was turned on. It lasted 20 ms and started 7 ms before the probe tone started. The frequency and intensity of the masking tone were varied while ABR signals were recorded. Again, 260 repetitions were averaged for each intensity/frequency combination.

Two data sets were measured for each mouse. One data set assessed the apical region using a 12 kHz probe tone and masking tones that ranged from 4–30 kHz. The other data set assessed the basal region using a 32 kHz probe tone and masking tones that ranged from 15–45 kHz. For both data sets, the stimulus intensity ranged from 10 to 80 dB SPL with 10 dB steps. When the masking frequency became close to the probe frequency, the protocol switched to using 5 dB intensity steps in order to provide better resolution in this critical region.

As with the ABR and DPOAE measurements, mice were anesthetized using ketamine (100 mg/kg) and xylazine (10 mg/kg). However because it took about two hours to test each mouse, we left a butterfly needle connected to the syringe containing the anesthetic inside the peritoneum to be able to provide supplementary anesthetic periodically during the experiment without disturbing the measurement.

Processing of each data set was performed offline to develop tuning curves. At each frequency and stimulus intensity combination, the ABR signal was analyzed to determine the N1/P1 voltage. The threshold at each frequency was determined to be the point at which the N1/P1amplitude dropped to 50% of the maximum and above 5 standard deviation of the noise floor. If ABR was detected even at our equipment limits, we arbitrarily defined the threshold to be 80 dB SPL. Finally, the tuning curve sharpness (Q_10dB_) was computed by dividing the bandwidth measured 10 dB above threshold by the probe frequency.

### Whole-mount preparations

Excised cochleae were fixed in 4% paraformaldehyde at room temperature for 1 hour and then immersed in 0.5 M EDTA for overnight. They were then rinsed three times (5 minutes per rinse) in phosphate-buffered saline containing 0.1% Triton-X100 (PBST) at pH 7.4. The entire length of the organ of Corti was then dissected free of each cochlea under a microscope.

The organ of Corti was immersed in a blocking solution of 4% donkey serum (017-000-121, Jackson Immuno Research Laboratories, West Grove, PA) in PBST for 1 hour at room temperature and then incubated with the primary antibody at 4°C overnight. Specimens were washed three times with PBST and then incubated with the secondary antibody at room temperature for 1 hour. The primary antibodies were rabbit anti-myosin VIIa (1∶200; Proteus Biosciences Inc., Ramona, CA) and goat anti-prestin N-20 (1∶500; Santa Cruz Biotechnology, Santa Cruz, CA). The secondary antibodies were Alexa Fluor 488 donkey anti-rabbit and Alexa Fluor 563 donkey anti-goat (1∶500; Invitrogen, Grand Island, NY).

After washing with PBS, specimens were mounted in anti-fade Fluorescence Mounting Medium (DAKO, Carpinteria, CA) and cover slipped. Images were acquired with a Zeiss LSM5 Pascal system using a 20X/0.5 EC Plan-NEOFLUAR ∞/0.17 objective. Seventeen or eighteen images were captured from each cochlea. At each location, the focus was adjusted to image either the OHCs or the IHCs, and the filter set changed to collect either the green or the red channel. The complete length of the cochlea was carefully reconstructed by overlapping the common cells at the edges of the individual images in Photoshop (Version 11.0, Adobe System Inc, San Jose, CA). Cytocochleograms were then made by counting all inner and outer hair cells and clustering them into 20 different locations relative to their distance from the base of the cochlea, using 5.72 mm as the average length of a CBA/CaJ mouse [27].

### Prestin immunolabeling to measure OHC prestin-containing membrane surface area

Excised cochlea was fixed in 4% paraformaldehyde at room temperature for 1 hour. The apical bone of the cochlea was opened and the Reissner's membrane was removed to reveal the hair cells. The cochleae were then rinsed three times (5 minutes per rinse) in PBS containing 0.1% Triton-X100 (PBST) at pH 7.4. Immunolabeling in the cochlea for prestin was performed as described above. The goat anti prestin1:200 (Santa Cruz Biotechnology, Santa Cruz, CA) was used as primary antibody and Alexa Fluor 563 donkey anti-goat 1∶500 was used as secondary antibody (Invitrogen, Grand Island, NY). After washing with PBST again, the cochleae were glued upright in a chamber and imaged using a custom-built upright two-photon microscope [28]. We used 20X water immersion objective lens with an excitation wavelength of 800 nm to collect the prestin signals. Z-stacks from both the apical and middle regions of the cochlea were captured. Then, the apical turn of the cochlea was dissected off and two more Z-stacks were collected from basal turn of the cochlea. 3D reconstruction of the Z-stacked images was performed using Volocity software (v5.3.0, PerkinElmer, MA, USA). The diameters of the top and the bottom of the prestin immunolabeling in each OHC, as well as the length of each OHC, were measured. The radius was calculated to be the average of the top and bottom radii divided by two. Thus, the prestin-containing membrane (PCM) surface area for each OHC was 2π*radius*length. Finally, the average PCM surface area per OHC was calculated by averaging the PCM for all measured OHCs.

### Quantitative Real-Time PCR

We measured prestin, myosin VIIa, and GAPDH mRNA using qRT-PCR. Two cochleae from each mouse were quickly dissected in cold PBS and placed in lysis buffer (RNAqueous Micro-Kit, AM1931, Invitrogen). The cochleae were homogenized in a 1.5 ml microcentrifuge tube using a pestle motor mixer (47747–370, VWR) for 1 minute on ice. The RNA was then isolated, treated with DNase I, and purified using the same kit as above. The samples were assayed for RNA concentration and purity using a Nanodrop ND1000 spectrophotometer (Thermo Scientific, Wilmington, DE, USA). Samples not meeting concentration (<150 ng/µl) and purity (A260/A280≥1.8) criteria, were discarded. cDNA was synthesized using iScript Reverse Transcription Supermix for RT-qPCR (170–8841, Bio-Rad).

We selected and optimized primers for target amplification (**Table 1**). The relative gene expression was measured using SsoFast^TM^ EvaGreen® Supermix (175–5204, Bio-Rad) in the thermal cycler (CFX96 Real-Time System, Bio-Rad). Each PCR reaction contained: 1 µl cDNA, 10 µl SsoFast EvaGreen Supermix, 2 µl primers and 7 µl MilliQ water. In order to quantify the relative gene expression in noise-exposed versus control mice, the mean of threshold cycle (C_T_) from the PCRs for the target genes and control gene (GAPDH) were compared using the 2^−(ΔCT)^  = 2^−(CTtarget-CTinternal control)^ relative quantification method [29].

### Western blot

Each isolated cochlea was placed in a 1.5 ml microcentrifuge tube with lysis buffer (50 mMTris-HCL pH 8, 150 mM sodium chloride, 1% triton X-100, 1 mM phenylmethylsulfonyl fluoride (PMSF), 1X protease inhibitor cocktail (cOmplete Mini, EDTA-free, 11836170001, Roche) and homogenized with a pestle motor mixer (47747-370, VWR) for 30 seconds on ice. Samples were then centrifuged at 13200 rpm for 5 minutes at 4°C (Eppendorf Centrifuge, 5415R). The supernatant was extracted and placed into a new microcentrifuge tube, homogenized again for 30 seconds, and kept on ice for 20 minutes. The supernatant was then centrifuged at 13200 rpm for 20 minutes at 4°C and the pellet was collected for further analysis.

The protein concentration of each sample was calculated by comparing the absorbance at 562 nm with gradient diluted standards of bovine serum albumin (BSA) (Pierce BCA Protein Assay Kit, 23227, Thermo Scientific). The sample concentrations were diluted to 980 µg/ml with buffer containing 50 mM Tris-HCL pH 8, 150 mM sodium chloride, and 1% triton X-100. Samples were then mixed with Laemmli sample buffer (161–0737, Bio-Rad) at a 2∶1 ratio, heated at 95°C for 5 minutes, cooled on ice, and loaded into precast gels (Mini-Protean TGX, 4–15%, 456–1085, Bio-Rad). The gel electrophoresis lasted 30 minutes at 200 V and then the samples were electrophoretically transferred to polyvinylidene fluoride (PVDF) membranes (Immobilon-FL, Millipore) at 30 V overnight at 4°C.

The membranes were washed with 1X Tris-buffered saline with 0.1% Tween 20 (TBST) three times (5 minutes per rinse) before blocking with 5% non-fat milk overnight at 4°C on a shaking platform. The membrane was then incubated with goat anti-prestin (1∶200, N-20, Santa Cruz), rabbit anti-Myosin VIIa (1∶1000, Proteus), and rabbit anti-GAPDH (1∶1000, 14C10, Cell Signaling) at 4°C overnight followed by three 10-minute washes in TBST. Secondary antibodies with HRP conjugates were added: donkey anti-goat (1∶2000, SC-2020, Santa Cruz) and goat anti-rabbit (1∶3000, 170-6515, Bio-Rad) for 1 hour at room temperature with shaking. The membranes were washed three times for 10 minutes each in TBST and treated with Immun-Star WesternC Chemiluminescent Kit (Bio-Rad) before detected by Fluor ChemQ (Alpha Innotech). Using Alphaview SA image analysis software (Alpha Innotech), the prestin, myosin VIIa, and GAPDH bands were selected using a box of constant size for every blot and their densities analyzed.

### Statistical analyses

Statistical analyses were performed using Excel (Microsoft, Seattle, WA), SPSS (IBM), and SigmaPlot (Systat Software Inc.). All values in figures are presented as mean ± SEM. Comparisons of averaged data were performed using the one-way ANOVA for three categories and the paired or unpaired Student's t-test (as indicated) for two categories. Tests with multiple measures at multiple points (i.e. ABR thresholds, DPOAE thresholds, and CM magnitudes) were compared by two-way ANOVA. P-values <0.05 were considered statistically significant.

## Results

### Noise exposure causes hair cell loss

**Figure 1:**
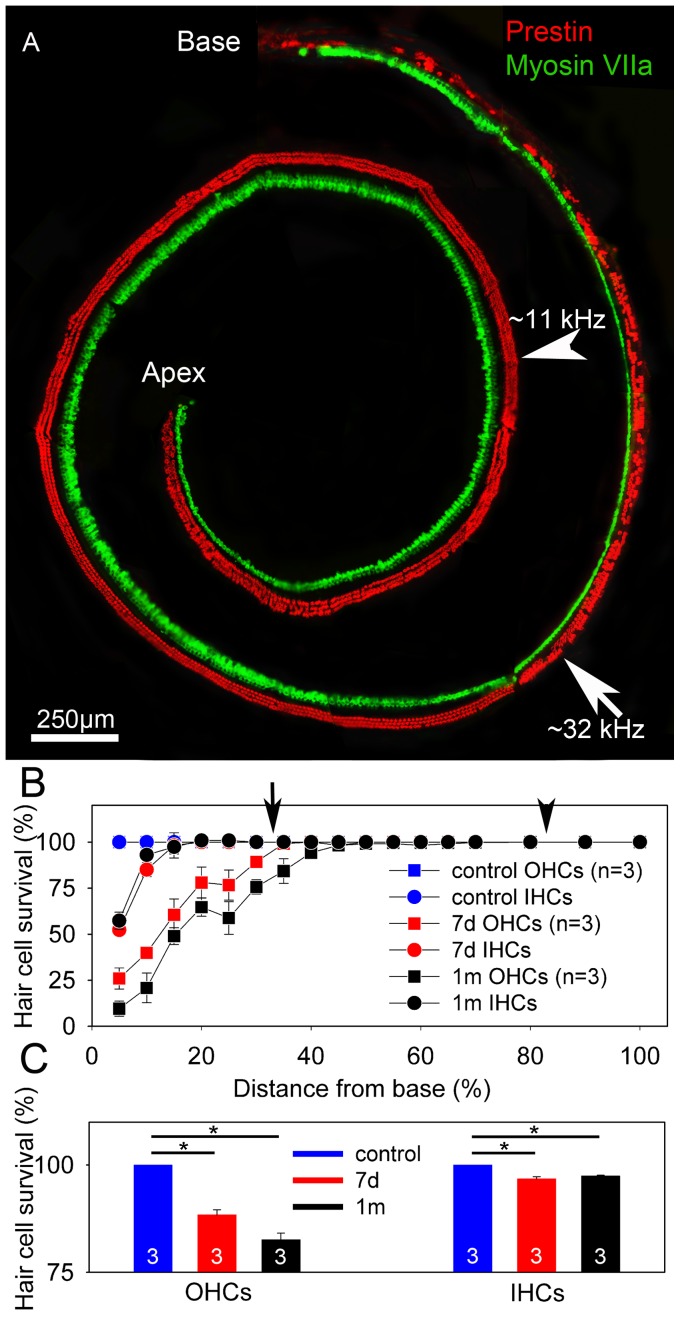
Noise exposure causes hair cell loss in the cochlear base. (**A**) One representative whole-mount cytocochleogram from a mouse cochlea harvested seven days after noise exposure is shown. Z-stacks were collected and summed using confocal microscopy. Prestin (*red*) labels OHCs. Myosin VIIa (*green*) labels both IHCs and OHCs, although we only summed the z-sections containing the IHCs for this image to distinguish them from OHCs. There was partial loss of hair cells in the basal region of the cochlea, whereas there was no hair cell loss in the apical region. The arrowhead highlights the 11 kHz region and arrow highlights the 32 kHz region. (**B**) The cytocochleogram counts revealed that more OHCs were lost than IHCs, and there was a gradient, with the most loss at the extreme cochlear base. (**C**)Total OHCs numbers progressively declined at 7 days and 1 month after noise exposure, whereas IHC numbers stabilized 7 days after noise exposure.

The noise exposure protocol was applied to cohorts of mixed-sex adult CBA/CaJ mice that were 5–6 weeks old. To assess how many hair cells were lost, we performed cytocochleograms seven days and one month (29–32 days) after noise exposure (three cochleae were in each cohort). Immunolabeling of prestin and myosin VIIa was performed and images were collected along the entire length of cochlea by confocal microscopy (**Fig 1A**). Hair cell loss was found predominantly within the basal third of the cochlea and there was no hair cell loss in the apical half of any cochlea.

We counted the number of missing and surviving hair cells in each mouse. In order to take into account the potential for differences in cell counts due to dissection variability between mice, we summed the number of missing and surviving hair cells together to get the total number of potential hair cells for each mouse. We then used this total to normalize the number of surviving hair cells and get the hair cell survival rate (**Fig 1B**). Seven days after noise exposure, there were 2086±18 OHCs and 729±6 IHCs, compared to the potential of 2360±20 OHCs and 753±7 IHCs. One month after noise exposure, there were 1916±19 OHCs and 747±7 IHCs, compared to the potential of 2320±20 OHCs and 768±7 IHCs. Thus, 88% of OHCs survived seven days after the noise exposure (unpaired t-test, p<0.001) and 83% survived one month after the noise exposure (unpaired t-test, p<0.001) (**Fig 1C**). The progressive loss of OHCs between seven days and one month after noise exposure was also significant (unpaired t-test, p = 0.04). IHCs fared better, with 97% of IHCs surviving both seven days and one month after the noise exposure (unpaired t-test, p = 0.03) (**Fig 1C**). Finally, the percent loss of OHCs was greater than the percent loss of IHCs at both time points (unpaired t-tests, 7 days: p = 0.003, 1 month: p<0.001). Together, these data demonstrate that our mouse model reliably produced a level and pattern of hair cell loss consistent with what others have found using a similar noise exposure protocol [30].

### Patch clamping indicates increased functional prestin in residual OHCs

**Figure 2:**
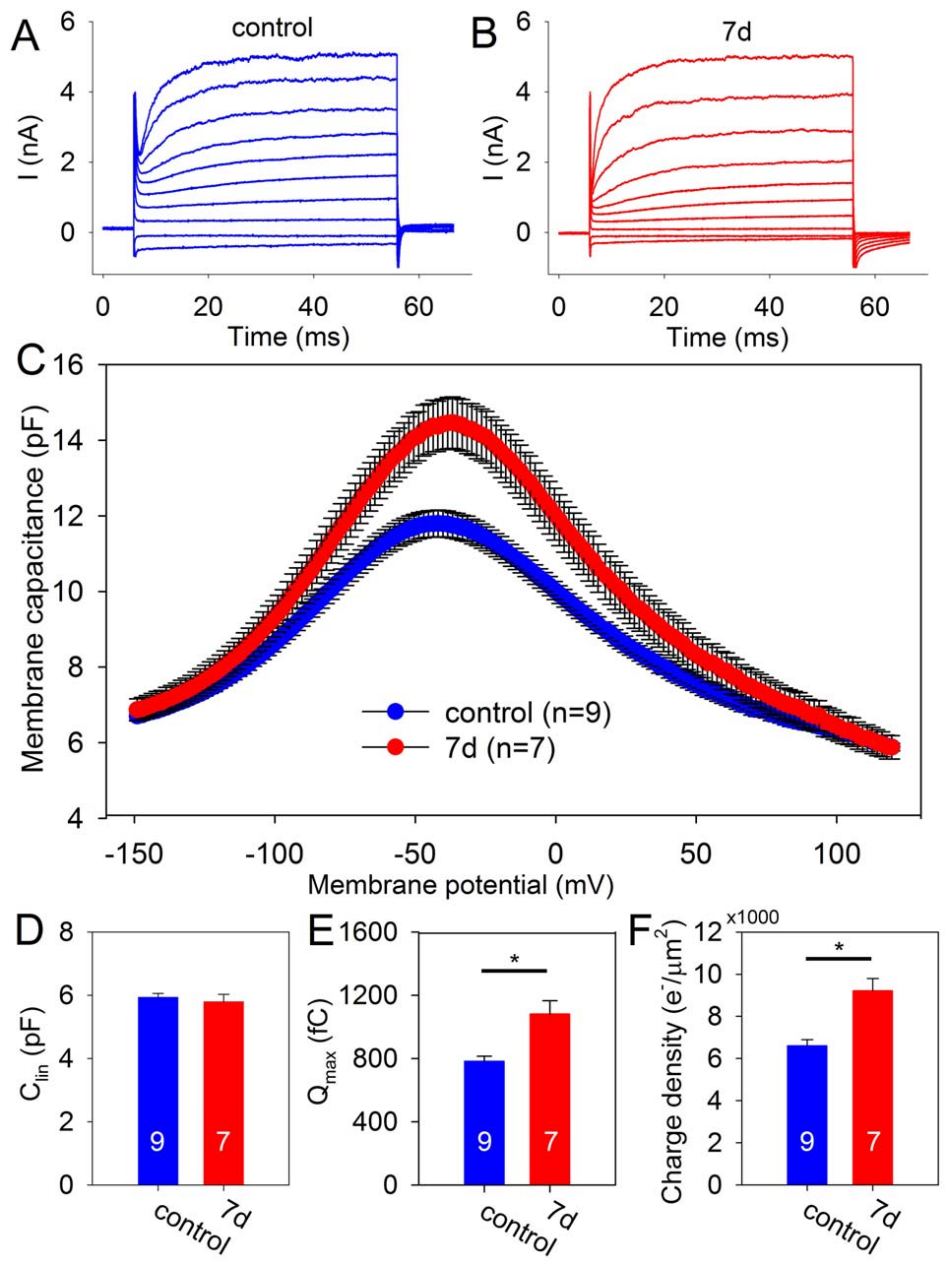
Functional prestin increases after noise exposure. (**A,B**) Current tracings in response to voltage steps from −100 mV to +80 mV in 20 mV steps from representative OHCs from control mice and mice 7 days after noise exposure. (**C**) Average capacitance as a function of voltage from control and noise-exposed mice. The non-linear capacitance was significantly higher in OHCs 7 days after noise exposure. (**D**) The linear capacitance of OHCs from control and noise-exposed mice were not significantly different. (**E**) The total charge moved (Q_max_) was greater in OHCs from noise-exposed mice. (**F**) The charge density was greater in OHCs from noise-exposed mice.

Next, we evaluated functional prestin levels in residual OHCs using the patch clamp technique. We dissected the organ of Corti from the apical turn of mice seven days after noise exposure as well as in control mice. The tectorial membrane was carefully removed prior to patch clamping. For consistency, we only patched first row OHCs about a half-turn down from the helicotrema. Cells that appeared unhealthy (i.e. swollen or containing intracellular particles with substantial Brownian motion) were not studied. Altogether, we collected data from nine control and seven noise-exposed OHCs. After entering the whole-cell configuration, we measured currents in response to a voltage step protocol to determine cell health. OHCs from both noise-exposed and control mice demonstrated typical membrane currents (**Fig 2A,B**) [31]. The average resting potential of the two groups of cells were identical (−76±4 mV for noise-exposed and −69±5 mV for control; unpaired t-test, p = 0.2).

Next, the two-sine wave technique was used to measure the capacitance over the voltage range of −150 to +120 mV (**Fig 2C**). This demonstrated that the peak capacitance in noise-exposed mice was greater than in controls (control: 11.9±0.3 pF; 7 days: 14.4±0.6 pF; unpaired t-test, p = 0.002). However, the voltage at peak capacitance was similar between the two cohorts (control: −39±2 mV; 7 days: −35±1mV; unpaired t-test, p = 0.14).We fit the capacitance tracings with the first derivative of a Boltzmann function and calculated the linear capacitance, the total elementary charge movement (Q_max_), and the charge density [7], [32]. The linear capacitance of OHCs from noise-exposed and control mice were not significantly different (control: 5.9±0.1 pF; 7 days: 5.9±0.2 pF; unpaired t-test, p = 0.8) (**Fig 2D**). However, the Q_max_ was elevated after noise exposure (control: 782±32 fC; 7 days: 1081±84 fC; unpaired t-test, p = 0.003) (**Fig 2E**). Also, the charge density was elevated after noise exposure (control: 6604±283 e^−^/µm^2^; 7 days: 9210±589 e^−^/µm^2^; unpaired t-test, p<0.001) (**Fig 2F**). These data indicate that even though OHC size did not change (as based on the linear capacitance data), functional prestin levels increased in residual OHCs after noise exposure.

### Prestin mRNA changes

We then performed qRT-PCR to measure mRNA levels of prestin within the entire cochlea after noise exposure. Because we wanted to determine whether the amount of prestin mRNA per hair cell changed, the ideal internal reference gene would be one that is only found in OHCs and whose expression level does not change after noise exposure. Unfortunately no known gene meets these criteria. Thus, we used two different internal references and the data must be interpreted with an appropriate understanding of their meaning. The first reference gene was GAPDH, a commonly-used housekeeping gene that is present in all cells. However, since the number of OHCs is small relative to the total number of cells in the cochlea, its level will not drop significantly when some OHCs are lost after noise exposure. Nevertheless, since the cochlea is surrounded by bone and it is relatively easy to remove all extraneous skeletal muscle off of it, GAPDH estimates the health and total amount of mRNA in the cochlea. Thus, when used as a reference, it will provide a reasonable approximation of the total amount of prestin within the cochlea. The second reference gene was myosin VIIa, a protein that is found in inner and outer hair cells. Although myosin VIIa expression has been demonstrated to change after traumatic insult [33], the levels normalize again after a week. As such, myosin VIIa can serve as a surrogate marker to infer the total amount of residual hair cells in the cochlea.

**Figure 3:**
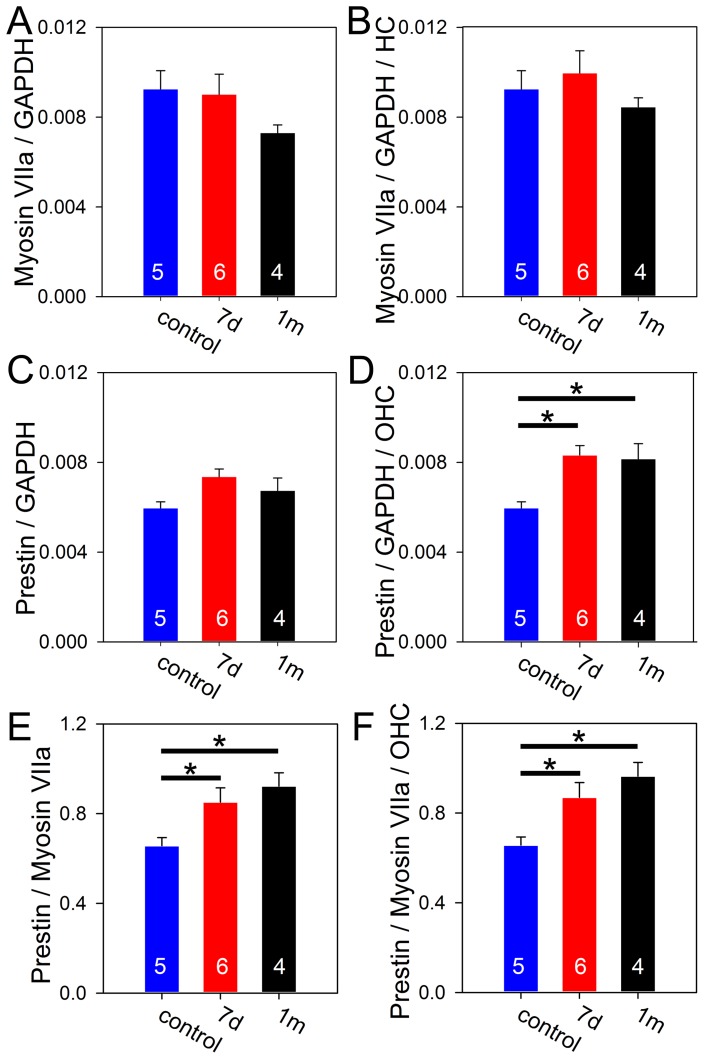
Prestin mRNA increases after noise exposure. (**A**) The total amount of myosin VIIa mRNA was not significantly different after noise exposure. (**B**) After normalizing myosin VIIa to the number of residual hair cells at 7 days and one month after noise exposure, there still was no difference in the amount of myosin VIIa per hair cell. (**C**) The total amount of prestin mRNA was not significantly different after noise exposure. (**D**) After normalizing prestin to the number of residual OHCs at 7 days and one month after noise exposure, there were statistically significant increases in the amount of prestin mRNA per OHC. (**E**) The prestin/myosin VIIa ratio increased after noise exposure. (**F**) After normalizing the prestin/myosin VIIa ratio to the number of residual OHCs, the increase after noise exposure persisted.

Data were analyzed in mice seven days after noise exposure, one month after noise exposure, and unexposed controls. There were no significant differences in the number of cycles to reach the amplification threshold for GAPDH with any of the cohorts (ANOVA, p = 0.4) demonstrating that our sample preparation was consistent. As expected, the myosin VIIa/GAPDH ratio and the prestin/GAPDH ratio were not significantly different between the cohorts (ANOVA, p = 0.29 and p = 0.91, respectively) (**Fig 3A, C**).

However, our hypothesis was that there should be more prestin per residual OHC, not more prestin within the entire cochlea. Therefore, we used the counts from the cytocochleograms to normalize the data. Since myosin VIIa is in every hair cell, the myosin VIIa/GAPDH ratio was divided by the proportion of residual inner and outer hair cells in each cohort (1 in controls, 0.90 at 7 days after noise, and 0.86 at 1 month after noise). This demonstrated that the myosin VIIa/GAPDH ratio per hair cell remained unchanged (ANOVA, p = 0.54) (**Fig 3B**), thus demonstrating the reliability of myosin VIIa as a control. Since prestin is only found in OHCs, the prestin/GAPDH ratio was divided by the proportion of residual OHCs in each cohort (1 in controls, 0.88 at 7 days after noise, and 0.83 at 1 month after noise). This demonstrated that the prestin/GAPDH ratio per OHC increased after noise exposure (control: 0.0063±0.0005; 7 days: 0.0083±0.0004; 1 month: 0.0082±0.0007; ANOVA, p = 0.017; unpaired t-tests, 7 day vs. control: p = 0.007, 1 month vs. control: p = 0.04) (**Fig 3D**).

Lastly, we used myosin VIIa as the reference and simply normalized prestin against it. The prestin/myosin VIIa ratio progressively increased after noise exposure (control: 0.66±0.04; 7 days: 0.85±0.06; 1 month: 0.92±0.06; ANOVA, p = 0.026; unpaired t-tests, 7 day vs. control: p = 0.04, 1 month vs. control: p = 0.005) (**Fig 3E**). After further normalizing prestin against the proportion of residual OHCs and myosin VIIa against the proportion of residual total hair cells, the prestin/myosin VIIa up-regulation per OHC was even larger (control: 0.66±0.04; 7 days: 0.87±0.07; 1 month: 0.96±0.06; ANOVA, p = 0.014; unpaired t-tests, 7 day vs. control: p = 0.03, 1 month vs. control: p = 0.003) (**Fig 3F**). Together, these findings support the concept that prestin is up-regulated after noise exposure.

### Prestin protein changes

**Figure 4:**
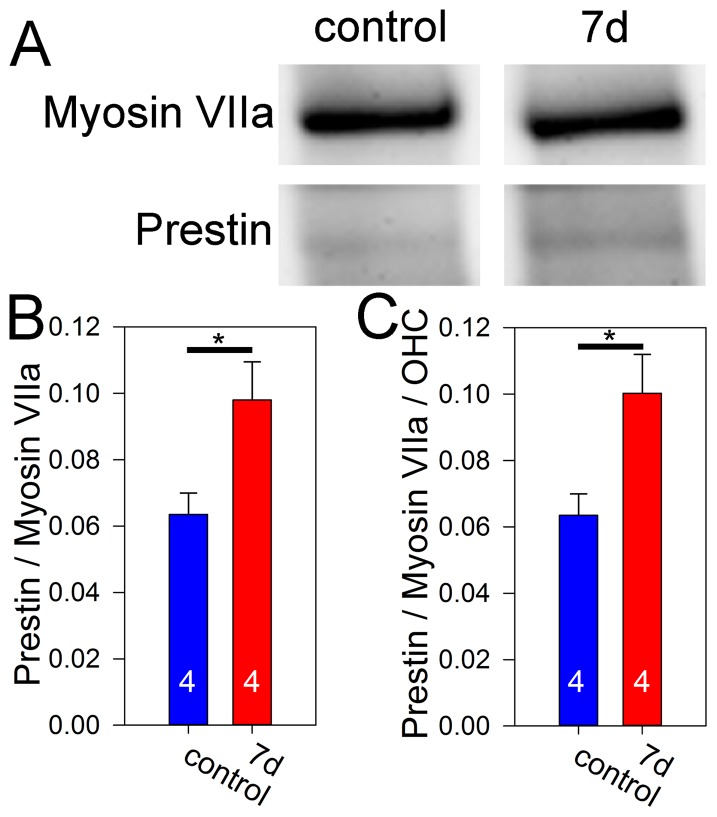
Prestin protein level increases after noise exposure. (**A**) Representative Western blots of prestin and myosin VIIa from mice seven days after noise exposure and from control mice. These images come from the same gel. (**B**) Quantification of the band density revealed that the prestin/myosin VIIa ratio increased after noise exposure. (**C**) After normalizing the prestin/myosin VIIa ratio to the number of residual OHCs, the increase after noise exposure persisted.

We next performed western blot studies to measure the amount of prestin protein within the entire cochlea. We compared four mice 7 days after noise exposure to four age-matched control mice. Each sample was repeated three times and the results averaged. Prestin had substantially weaker bands relative to the easily visible myosin VIIa (**Fig 4A**). First, we directly normalized prestin band density to the myosin VIIa band density and found that the prestin/myosin VIIa ratio increased after noise exposure compared to controls (control: 0.063±0.006; 7 days: 0.098±0.011;unpaired t-test, p = 0.04) (**Fig 4B**). We then extended the analysis, similar to how we analyzed the qPCR data, in order to take into account the missing hair cells based on the cell count data. Thus, each prestin band density from a noise-exposed mouse was divided by 0.88 (the number of remaining OHCs) and each myosin VIIa band density from a noise-exposed mouse was divided by 0.90 (the proportion of remaining hair cells, both IHCs and OHCs). In this case, the prestin/myosin VIIa ratio was even greater (control: 0.063±0.006; 7 days: 0.100±0.011; unpaired t-test, p = 0.03) (**Fig 4C**). Like the qPCR data, these findings are consistent with the concept of prestin up-regulation in hair cells remaining after noise exposure.

### Prestin prediction model

In our measurements of prestin mRNA and protein levels using whole cochlea specimens described above, we normalized them to the number of residual OHCs. While this technique was helpful in providing an initial interpretation of the data, it was not as comprehensive of a model as we desired because, OHC size and the amount of prestin in each OHC vary along the length of the cochlea [11]. Thus, removing small OHCs at the base while leaving large OHCs at the apex, as our noise exposure protocol does, would raise the average prestin per OHC even if the actual amount of prestin per residual OHC did not change. To determine whether this effect was behind the increased prestin levels we found, we created a model to predict its impact.

**Figure 5:**
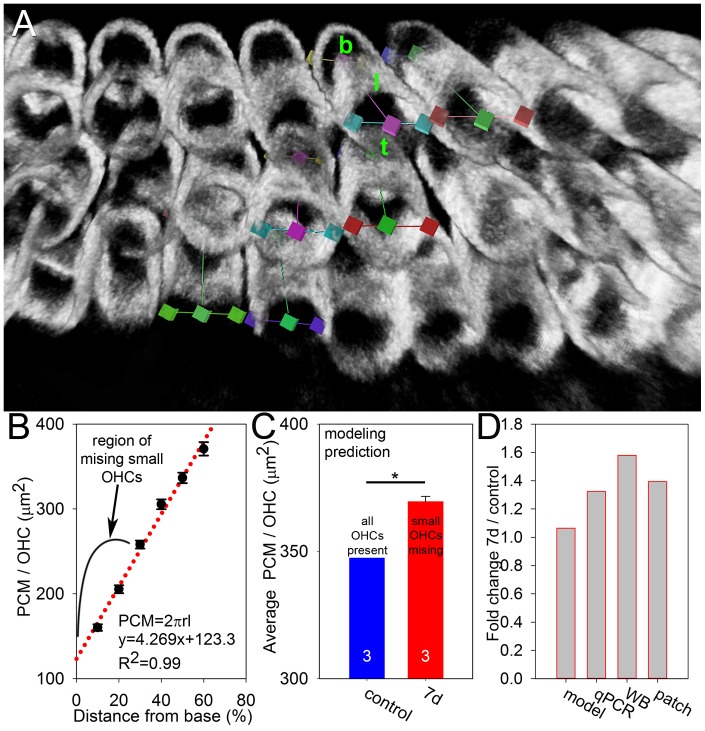
The measured prestin increases are greater than modeling predictions. (**A**) Representative whole-mount preparation immunolabeled for prestin imaged by two-photon microscopy. We measured the diameter of the OHC at the top (*t*), the diameter of the OHC at the bottom (*b*), and the length of the OHC (*l*) to calculate the amount of prestin-containing membrane (PCM) per OHC. (**B**) The PCM/OHC measured at six different cochlear locations demonstrated a linear relationship. The region where the small OHCs from the base that were lost after noise exposure is shown. (**C**) The data from the cytocochleograms were used to predict what would happen, assuming the amount of prestin per OHC does not change and only the smaller OHCs from the base are removed from the average. According to this model, a statistically significant increase in the PCM/OHC should be expected. (**D**) However, the fold-increases in prestin we measured by qPCR and Western blot were larger than that predicted by the model. For reference, the fold-increase in the functional prestin density measured with the patch clamping experiments is also shown.

First, we measured the amount of prestin containing membrane (PCM) surface area in control cochlea at six different locations. Excised cochlea were studied with the epithelium left *in situ*, with only the overlying otic capsule bone opened to permit prestin immunolabeling followed by imaging with two-photon microscopy. Thus, dissection artifacts were substantially minimized. Reconstruction of the Z-stack images was performed in 3D and the diameters at the top of each OHC, the diameters at the bottom of each OHC, and the lengths of each OHC were measured (**Fig 5A**). The PCM surface area was calculated, plotted versus cochlear location, and a trend line was fit (**Fig 5B**).The average PCM per OHC in a control mouse was simply the average of this trend line over the entire length of the cochlea (*first bar,* **Fig 5C**). The average PCM per OHC after in a noise-exposed mouse was estimated by using the data from the cytocochleogram seven days after noise exposure to subtract out missing OHCs from their appropriate positions. Since only the small, basal cells were removed, this raised the average PCM per OHC by 6.4±0.6% (*second bar,* **Fig 5C**). Therefore, even if there was no change in the amount of prestin in any given OHC, the model predicted that the average prestin per OHC would be greater in mice 7 days after noise exposure compared to controls (unpaired t-test, p<0.001).

We then compared the prestin fold change we measured using qPCR and Western blot against the model's prediction (*first three bars*, **Fig 5D**). This demonstrated that the prestin per OHC increased more than would be expected simply by the elimination of smaller OHCs. Prestin mRNA was 32% higher and prestin protein was 58% higher than controls. For comparison, the actual level of functional prestin density measured by the patch clamp studies was 39% higher than controls (**fourth bar**, **Fig 5D**). Together, these data demonstrate that the amount of prestin per residual OHC increases between 32–58% after noise exposure, and that this is not simply due to removing small OHCs from the average.

### Consequences of noise exposure on cochlear function

**Figure 6:**
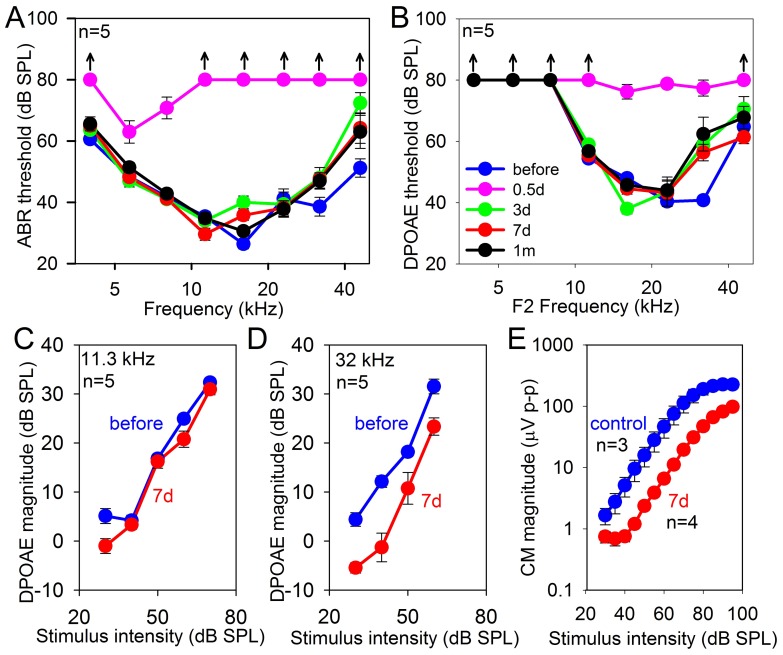
ABR, DPOAE, and cochlear microphonic measurements. (**A, B**) ABR and DPOAE thresholds were dramatically elevated 0.5 days after noise exposure. There was complete recovery in the low frequencies and partial recovery in the high frequencies. (**C**) DPOAE growth curves demonstrate similar magnitude emissions between noise-exposed and control mice at 11.3 kHz, the area where we performed the patch-clamp studies. (**D**) At 32 kHz, a region where there was partial OHC loss, noise-exposed mice had lower DPOAE magnitudes. (**E**) The cochlear microphonic magnitude measured at the round window using a 6 kHz stimulus was lower in noise-exposed mice compared to controls.

To assess the effect of our noise exposure mouse model on auditory function, we measured auditory brainstem responses (ABRs) and distortion product otoacoustic emissions (DPOAEs) within a cohort of five mice (five ears). We serially tested the mice before noise exposure (control) and again at 0.5 days, 3 days, 7 days, and 1 month after noise exposure (**Fig 6A, B**). ABR and DPOAE thresholds were substantially elevated over the whole frequency spectrum 0.5 days after the noise exposure (two-way AVONA, p<0.001 for both ABRs and DPOAEs). By 1 month after the noise exposure, ABR and DPOAE thresholds had fully recovered at low frequencies (8–11.3 kHz) (two-way AVONA, p = 0.882 and p = 0.133, respectively), whereas they had only partially recovered at high frequencies (31.9–46 kHz) (two-way AVONA, p = 0.015 and p = 0.03, respectively). These data are consistent with the concept of the noise exposure producing an initial temporary threshold shift that partially improves over time to a produce a lesser degree of permanent threshold shift [30]. This also fits with the pattern of hair cell loss we noted in the basal region of the cochlea.

Since non-linearities associated with cochlear amplification and prestin can be assessed with DPOAEs, we then looked at the 2F1–F2 amplitudes versus stimulus intensity. Our (perhaps) simplistic concept was that if prestin levels were increased, OHCs should generate more electromotility and thus the emission amplitude for any given stimulus intensity should be greater. We studied two frequency regions, 11.3 kHz and 32 kHz. While the exact site/sites of DPOAE generation remain questionable, these sites were selected because 11.3 kHz is a frequency region on the cochlear tonotopic map roughly where we performed the patch clamp studies and where there was definitely no OHC loss, and 32 kHz is a frequency region where there was mild OHC loss (*arrowhead and arrow in* *Fig 1A*, *respectively*). At 11.3 kHz, there was no difference between the growth curves of control and noise-exposed mice (two-way ANOVA, p = 0.2) (**Fig 6C**). At 32 kHz, noise-exposed mice had lower emission amplitudes (two-way ANOVA, p<0.001) (**Fig 6D**). Thus, while the loss of some OHCs reduced emission amplitudes, the addition of prestin to OHCs did not result in a measureable increase of emission amplitudes.

We also measured the cochlear microphonic by placing a silver wire on the round window membrane and measuring the electrical field potential during the application of a 6 kHz stimulus. This evaluates the compound sum of the receptor potentials of OHCs from the cochlear base (**Fig 6E**). The magnitude of the cochlear microphonic was reduced in noise-exposed mice compared to age-matched controls (two-way ANOVA, p<0.001). This finding is consistent with the pattern OHC loss at the base of cochlea that we noted earlier.

### Tuning curve measurements to assess cochlear amplifier function

The function of prestin is to drive the cochlear amplifier, increasing auditory sensitivity and sharpening frequency tuning [34]. To determine if the increased OHC prestin produced these effects, we used our custom optical coherence tomography (OCT) setup to measure basilar membrane vibratory tuning curves [24]. Using living, anesthetized mice, we imaged the organ of Corti about a half-turn down from the helicotrema. Although the bulla was opened to visualize the cochlea, the cochlea itself was not opened as our system can peer through the otic capsule bone. The optical path was aimed to cross the basilar membrane directly under the OHC region, and basilar membrane vibratory displacements were measured in response to auditory stimuli from 3–12 kHz using a stimulus intensity of 60 dB SPL. We normalized these vibrational magnitudes to the middle ear response measured from the orbicularis apophysis of the malleus.

**Figure 7:**
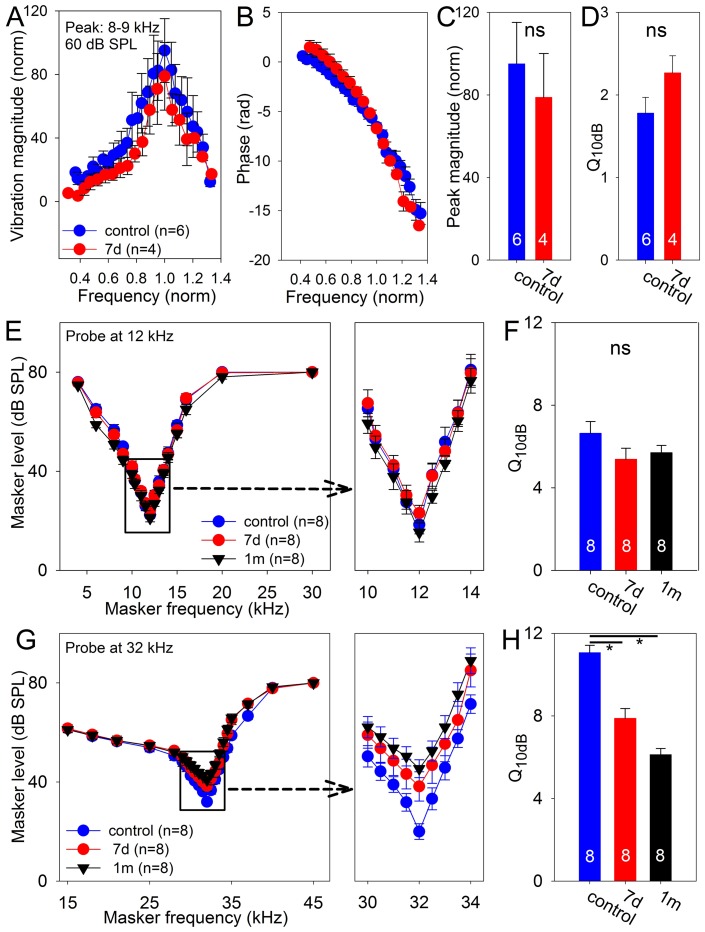
Cochlear tuning curves. (**A, B**) Basilar membrane vibratory magnitude and phase measured in the 8–9 kHz region using a 60 dB SPL stimulus are shown. In order to allow averaging, the frequency was normalized so that the resonance frequencies were 1.0, and the magnitudes was normalized to the middle ear response. (**C**) There were no differences between in the peak magnitudes of tuning curves measured in noise-exposed and control mice. (**D**) There were no differences between in the sharpness of the tuning curves measured in noise-exposed and control mice. (**E**) CAP tuning curves collected serially in a cohort of mice using a 12 kHz probe tone. (**F**) There were no significant difference in the sharpness of the tuning curves. (**G**) CAP tuning curves were also collected using a 32 kHz probe tone. (**H**) The tuning curve sharpness dropped after noise exposure in this region where some OHCs were lost.

We studied six control mice and four mice 7 days after noise exposure. All mice demonstrated tuned responses with the resonance frequency peak magnitude at 8.9±0.3 kHz. We normalized the stimulus frequencies to center the peak resonances and then averaged the curves (**Fig 7A**). Both cohorts of mice demonstrated similar basilar membrane vibratory magnitudes. There were no differences between their peak vibratory magnitude ratios (control: 95±20; 7 days: 79±21; unpaired t-test, p = 0.6) or their Q_10dB_ values (control: 1.8±0.2; 7 days: 2.3±0.2; unpaired t-test, p = 0.13) (**Fig 7C, D**). Phase responses were similar between the cohorts as well, and demonstrated the characteristic phase delay with increasing frequency consistent with traveling wave propagation (**Fig 7B**).

To confirm these results and to test a high frequency cochlear region where our OCT system is unable to record from, we also measured masked compound action potential (CAP) tuning curves [25], [35]. To maintain consistency with the DPOAE growth curves we previously measured, we selected probe frequencies of 12 and 32 kHz. Recordings were made using scalp needle electrodes, similar to how ABRs are recorded, so that repeated measurements could be made in the same animals at multiple time points. We recorded tuning curves from one cohort of eight mice before noise exposure, 7 days after noise exposure, and 1 month after noise exposure. Using the 12 kHz probe tone, the tuning curves were similar (**Fig 7E**). We calculated the Q_10dB_ at each measurement time point and found no differences (control: 6.6±0.6; 7 days: 5.4±0.5; 1 month: 5.7±0.3; ANOVA, p = 0.2) (**Fig 7F**). These data are consistent with the mechanical measurements made above. In contrast, the tuning curves demonstrated less sensitivity and lower Q_10dB_ levels when using the 32 kHz probe tone (control: 11.0±0.4; 7 days: 7.9±0.5; 1 month: 6.1±0.3; ANOVA, p = 0.025; paired t-test 7 day vs. control: p<0.001, 1 month vs. control: p<0.001) (**Fig 7G,H**). This progressively worsened from 7 days to 1 month after noise exposure (paired t-test, p = 0.02), consistent with our finding of progressive loss of OHCs during this time point (see Fig 1). It is conceivable that degeneration of spiral ganglion neurons may also play a role in this effect [36].

## Discussion

In this study, we used a noise exposure protocol to cause hair cell loss localized to the basal region of the cochlea that produced high frequency hearing loss. This caused residual OHCs to increase their expression of prestin mRNA and protein. Since prestin is directly linked to voltage-dependent OHC length changes (electromotility), this may be one mechanism by which the cochlea can compensate, at least partially, for hearing loss. It seems logical that in a cochlear region where there is sporadic loss of OHCs (closer to the base), having the adjacent OHCs produce more electromotile force could be beneficial. However in a region where all the hair cells were present (closer to the apex), increased prestin did not result in supra-normal auditory sensitivity or frequency tuning. We conclude, therefore, that additional homeostatic mechanisms function specifically to modulate how OHC force production effects changes in the vibration of the cochlear partition. Thus, the gain of the cochlear amplifier is clearly not simply a function of OHC prestin level or the non-linear capacitance: it is actively-regulated.

Previous studies of noise exposure have shown an increase of prestin mRNA in OHCs after noise exposure but did not investigate prestin protein expression levels or prestin function. One group showed that rats exposed to long periods of noise had profound OHC loss and a nearly 5-fold increase in prestin mRNA after 5 days, followed by a return to the baseline by 4 weeks [37]. Other group also found 3 to 4 fold increase of prestin mRNA in the noise exposed cochleae [38]. Prestin protein and mRNA were investigated in the chronic salicylate administration induced hearing loss [39]. They found an up-regulation of prestin mRNA, as well as protein, after ototoxic drug administration. Together with our data in noise-exposed mice and our previous finding of prestin increases in Tecta^C1509G^ transgenic mice, these data support the concept that prestin up-regulates as a general response to hearing loss.

There are several potential sources that may provide feedback to the OHC to regulate prestin levels and/or maintain homeostasis in the face of changes in prestin levels. One possibility that we consider unlikely is central feedback through efferent pathways. Mice in which the alpha9 acetylcholine receptor on OHCs has been knocked out do not demonstrate altered auditory thresholds from wild-type mice [40], [41]. Moreover, alpha9 null mice do not demonstrate changes in DPOAEs or electromotility [42]. Noise exposure, however, does demonstrate differences between the alpha9 null mice and wild-type mice [41], [43]. Nevertheless, it is somewhat surprising that there appear to be no functional differences in baseline auditory thresholds even though the efferent neurotransmitter, acetylcholine, hyperpolarizes OHCs and reduces the gain of the cochlear amplifier [44]–[47]. One possible explanation of this finding is that the same homeostatic mechanism that compensates for increased prestin levels after noise exposure is also compensating for the lack of efferent input. It is, however, conceivable that non-cholinergic neurotransmitters in the efferent system [48], such as GABA [49], [50], may be responsible for this effect.

Local feedback mechanisms are potential contributors to maintaining the gain of the cochlear amplifier, regulating both prestin levels and perturbations in OHC force production. For example, prestin mRNA and protein are reduced in the absence of thyroid hormone during development [51]. As well, other transcription factors have been reported to play roles in regulating prestin expression, such as retinoid nuclear transcription factor, GATA-3, and Pou4f3 [52], [53]. OHC-specific analysis of gene expression after noise exposure will be needed to understand the signaling pathways involved in prestin regulation in adult animals.

Given that the organ of Corti is dedicated to tuning the physical stimulus reaching the inner hair cell stereociliary bundles, it is not unreasonable to consider that the mechanical properties of the highly-intertwined cells may also feedback on each other. For example, we do not know why the OHCs and Deiters' cells form a unique angled arrangement and interlock with one another. Could changes in the force production by the OHC lead to alterations of the stiffness of the Deiters' cell? Could pillar cell stiffness or the deformability of Hensen cells be modulated to fine tune cochlear mechanics? The study of cell-to-cell mechanical interactions is particularly critical within the cochlea, where vibratory patterns depend almost completely upon the mechanical impedance set by the cells, the acellular tectorial membrane and basilar membrane, and their interactions. *In vivo* studies of the vibratory patterns in situations of normal and altered cochlear anatomy may prove helpful in this type of analyses [54].

Once the feedback signal is provided to the OHC, how is prestin production regulated? Previously, we transduced prestin into immature OHCs using a constitutively-active CMV promoter prior to the normal expression time of prestin [55]. We found that prestin density was similar to that found in normal adult wild-type OHCs. We used this fact to argue that native prestin levels may not be predominantly regulated at the level of transcription but instead post-transcriptionally regulated by the surface area of the lateral plasma membrane or lateral interactions with other prestin molecules [56]. However, there was a large variability in the transferred charge density associated with prestin function in our data (4,140±1,092 e^−^/μm^2^; mean ± SEM).The variability is even larger (from 4,000–10,000 e^−^/μm^2^) in the multiple reports from other groups who have made this measurement in normal adult wild-type OHCs [22], [57]–[60]. Thus, the modest changes in prestin density we have measured, which appear to be regulated at the transcription level because of the increase in prestin mRNA, could certainly have been overlooked in the previously reported data.

Electron microscopy reveals a tight packing of particles within the OHC lateral wall membrane [61], and these numbers correlate with physiological measures of non-linear capacitance, suggesting that each particle is a prestin molecule [62]. This suggests that the amount of prestin within the lateral wall cannot increase substantially. Our data support this notion as the prestin increase was relatively modest. Our previous findings that the level of prestin in the plasma membrane in HEK cells is under endocytic regulation [4] suggests that other mechanisms of post-translational regulation of prestin levels in the lateral wall might exist the OHC.

Lastly, the OHC can modulate its own force production without changing prestin levels [63]. Changing OHC turgor pressure by modulating the solute concentration within the cytoplasm can alter plasma membrane tension, and thereby alter electromotility [64], [65]. Modulating the cholesterol concentration within the plasma membrane has also been shown to shift the voltage dependency of electromotility [4]–[6]. Changing the ratios or charge of the phospholipids within the bilayer membrane could also alter electromotility [66]. Regulation of the intracellular chloride concentration, which is proportional to the non-linear capacitance and electromotility [7], could alter electromotility. Deiter cells and gap junctions can also modulate electromotility [67].Many further studies will be needed to isolate the mechanism(s) that play a direct role to actively-regulate the cochlear amplifier *in vivo*.
